# Weekend Effect and Predictors of Mortality for Patients Presenting to Emergency Department with COVID-19 Infection

**DOI:** 10.3390/jpm15090402

**Published:** 2025-09-01

**Authors:** Amteshwar Singh, Jerome Gnanaraj, Evani Jain, Japleen Kaur, Waseem Khaliq

**Affiliations:** 1 Department of Medicine, Johns Hopkins University School of Medicine, Baltimore, MD 21287, USAjgnanar1@jhmi.edu (J.G.); 2 Department of Internal Medicine, Sinai Hospital, Baltimore, MD 21215, USA; 3 Department of Internal Medicine, Virginia Commonwealth University, Richmond, VA 23298, USA; japleen0406@gmail.com

**Keywords:** COVID-19, mortality, emergency department

## Abstract

**Background:** Weekend presentation to the emergency department (ED) has been associated with increased morbidity and mortality in various clinical settings. However, the literature is scant whether such an effect exists for patients presenting with COVID-19 infection. Additionally, comparative analyses of mortality predictors in COVID-19 patients evaluated at the emergency department need further exploration. **Methods:** This retrospective cohort study examined factors associated with mortality among adult patients (aged ≥ 18 years) who presented with COVID-19 to the emergency departments of five hospitals within the Johns Hopkins Health System (combined capacity: 2513 beds) between March 1 and 4 May 2020. Data were extracted from electronic health records. Multivariable logistic regression was utilized to assess the relationship between mortality and a range of variables, including sociodemographic characteristics, clinical presentation, laboratory parameters, pre-existing comorbidities, and weekend versus weekday presentation. **Results:** Of the 2767 patients, 685 (25%) presented to the emergency department on weekends. Compared to weekday presenters, weekend patients were more likely to be hospitalized (64%), and these patients had a mean symptom duration of 5 days (SD ± 6). Weekend presenters also exhibited higher rates of clinical frailty, dehydration, hypoxia, and respiratory distress upon arrival. In multivariable logistic regression analysis adjusting for sociodemographic characteristics, clinical risk factors, and laboratory findings, independent predictors of increased mortality included absence of a primary care provider (OR 3.47; 95% CI: 2.37–5.07), peripheral oxygen saturation (SpO_2_) < 95% at presentation (OR 1.46; 95% CI: 1.001–2.12), and hyperglycemia (OR 2.13; 95% CI: 1.25–3.65). Notably, the presence of crackles on physical examination demonstrated a trend toward reduced mortality (OR 0.47; 95% CI: 0.24–0.92). **Conclusions:** While weekend presentation was associated with higher hospitalization rates among patients with COVID-19, it did not independently predict increased mortality. Absence of a primary care provider, hypoxia, and hyperglycemia at presentation emerged as strong, independent predictors of mortality in the ED setting. Race, gender, and obesity were not significantly associated with mortality in this cohort, warranting further investigation. These findings may support more effective triage and risk stratification strategies in current and future public health emergencies.

## 1. Introduction

Coronavirus disease 2019 (COVID-19), caused by the severe acute respiratory syndrome coronavirus 2 (SARS-CoV-2), has resulted in over 7.1 million deaths globally since the onset of the pandemic in December 2019 [1]. In the United States (U.S.) alone, more than 1.2 million deaths have been reported since 1 January 2020. As of 2025, the estimated case fatality rate in the U.S. is approximately 0.6%, with current weekly mortality ranging between 200 to 900 deaths, substantially lower than the peak of the pandemic in 2021, when weekly deaths reached approximately 25,000 [2]. This decline in mortality is likely attributable to widespread population-level immunity, achieved through both natural infection and vaccination, improved understanding of disease pathophysiology, the implementation of airborne precautions and masking strategies, and the availability of effective therapeutic interventions [3,4]. Nevertheless, COVID-19 continues to contribute to substantial morbidity and mortality, particularly among older adults. Individuals aged 75 years and older remain at the highest risk of death [5]. In addition to advanced age, several clinical variables have been associated with worse outcomes among hospitalized patients with COVID-19, including frailty, underlying cardiovascular disease, hypoxemia at presentation, and elevated inflammatory markers [6,7,8].

Despite extensive literature on COVID-19 outcomes, data examining the relationship between weekend emergency department (ED)/hospital admissions and COVID-19–related mortality remain limited [9,10,11]. The concept of increased mortality associated with weekend admissions, commonly referred to as the “weekend effect”, has been the subject of investigation for several decades [12]. This phenomenon is hypothesized to stem from reduced hospital staffing, limited availability of diagnostics and specialist services, and constrained access to certain interventions during weekends compared to weekdays, although the precise mechanisms remain unclear [13]. A 2017 meta-analysis reported a 17% higher mortality rate for patients admitted during weekends compared to weekdays [14], while a subsequent meta-analysis in 2019 identified an 11% increase in mortality among patients with urgent admissions over the weekend [15]. This effect persists even after adjusting for the disease severity, suggesting that temporal factors independent of baseline clinical status may contribute to the observed disparities [16]. Evidence also indicates that the weekend effect may be mitigated through the allocation of additional resources aimed at expediting diagnosis and treatment [17]. Another potential explanation of the weekend effect is that limited access to outpatient care over the weekend results in delayed presentations and a higher acuity threshold for emergency admissions during this period [18].

This study aims to examine the ‘weekend effect’ on mortality among patients with COVID-19 who presented to the emergency department (ED) on weekends compared to weekdays. Additionally, we investigate predictors of mortality in this population, including socioeconomic factors, clinical presentations, and laboratory findings.

## 2. Methods

### 2.1. Study Design and Sample Size

This retrospective descriptive study included all adults (≥18 years) with COVID-19 infection, defined by a positive SARS-CoV-2 nucleic acid test and corresponding ICD-10 codes [19], who presented to emergency departments (EDs) within the Johns Hopkins Health System between 1 March and 4 May 2020, prior to the availability of COVID-19 vaccines. Clinical and demographic data were extracted from electronic health records across five hospitals within the system (total capacity: 2513 beds): the Johns Hopkins Hospital, the Johns Hopkins Bayview Medical Center, Howard County General Hospital, Suburban Hospital, and Sibley Memorial Hospital. Detailed methods for cohort identification and data extraction have been previously described [10]. In brief, patients were excluded if they were under 18 years of age, pregnant, or admitted primarily for psychiatric care. For individuals with multiple ED visits during the study period, only the first encounter was included in the analysis. The final study cohort comprised 2767 adult patients evaluated across the participating EDs of the Johns Hopkins Health care system. The study protocol was approved by the Institutional Review Board of the Johns Hopkins Health System.

### 2.2. Study Protocol, Variables, and Measures

A structured review of electronic medical records was conducted to extract relevant clinical and demographic data, including COVID-19 diagnosis, ED length of stay, day of presentation (with weekends defined as Saturday or Sunday), encounter type (ED visit versus inpatient admission), and hospital length of stay. Sociodemographic variables such as age, race, gender, ethnicity, marital status, and employment status were ascertained at the time of ED presentation, along with health behavior data including smoking status, alcohol use, and body mass index (BMI). Health care access was assessed based on insurance status and presence of having a primary care provider. Comorbidity burden was quantified using the Charlson Comorbidity Index (CCI), derived from ICD-10 diagnostic codes [20]. Clinical parameters, including presenting symptoms, vital signs, physical exam findings, laboratory results and imaging studies were recorded at the time of ED presentation. Discharge diagnoses were used to confirm final diagnoses when discrepancies existed for admission diagnosis. Data abstraction was performed using a standardized form by two independent reviewers, with discrepancies resolved by a third reviewer.

### 2.3. Statistical Analysis/Methods

Descriptive statistics were summarized as proportions and means for categorical variables. Unpaired *t*-tests and Chi-square tests, with statistical significance defined as *p*  ≤  0.05, were used to compare population characteristics, clinical signs and symptoms, vital statistics, laboratory and imaging workup, and admission diagnoses by weekday vs weekend status. A Fisher’s exact statistic was utilized where at least 20% of ex-pected cell frequencies were <5, whereas the Mann–Whitney U test was used where the data were not normally distributed. Univariable and multivariable logistic regression models were used to identify factors associated with mortality, including sociodemographic characteristics, clinical symptoms, comorbidities, and laboratory/imaging findings. Predictor variables were selected based on clinical relevance and the prior literature. Multicollinearity among covariates was evaluated using correlation matrices and the Variance Inflation Factor (VIF), with all individual VIF values < 2.2 and adjusted model variables mean VIF < 1.6, indicating no significant multicollinearity. Separate logistic models were constructed for each variable domain and subsequently adjusted for all relevant covariates in the final multivariable analysis. Statistical analyses were performed using Stata statistical software (StataCorp LP, Version 16.1).

## 3. Results

Among the 2767 patients who presented to the emergency department with confirmed COVID-19, 685 (25%) presented on weekends and 2082 (75%) on weekdays. The mean age of the study population was 55 years (SD ± 19); 34% identified as African American, and 33% reported Hispanic ethnicity. As shown in Table 1, baseline demographic and clinical characteristics were largely comparable between the weekend and weekday groups. However, patients presenting on weekends reported shorter symptom duration and were less likely to report recent travel. Notably, weekend presentations were associated with a higher likelihood of hospitalization. Although presenting symptom profiles and overall mortality rates were similar between groups (Table 2), patients presenting on weekends demonstrated a higher frequency of clinical severity markers, including frailty, dry oral mucosa, hypoxia, crackles on lung exam, and respiratory distress. No statistically significant differences were observed in primary admission diagnoses, laboratory parameters, or imaging findings between the two groups (Table 3).

As shown in Table 4, partially adjusted bivariate analyses identified several sociodemographic, clinical, and laboratory variables associated with mortality. In the fully adjusted multivariable logistic model, significant independent predictors of mortality included absence of a primary care provider (OR 3.47; 95% CI: 2.37–5.07), peripheral oxygen saturation < 95% (OR 1.46; 95% CI: 1.001–2.12), and hyperglycemia (glucose >100 mg/dL) at presentation (OR 2.13; 95% CI: 1.25–3.65). Conversely, the presence of crackles on physical examination was associated with a 53% lower likelihood of mortality (OR 0.47; 95% CI: 0.24–0.92) suggesting a possible protective effect.

## 4. Discussion

This study found that patients presenting to the ED with COVID-19 on weekends were more likely to be admitted to the hospital compared to those presenting on weekdays. However, no ‘weekend effect’ was observed in terms of mortality; outcomes were similar regardless of the day of presentation. Additionally, the study identified three independent predictors of mortality: absence of a primary care physician (PCP), hypoxia on admission, and hyperglycemia on admission. These findings contribute to the evolving understanding of COVID-19 outcomes during the early phase of the pandemic and underscore the importance of early recognition of high-risk features using personalized approach.

The observed increase in hospital admission rate for COVID-19 patients presenting on weekends in our study contrasts with the earlier literature suggesting higher admission rates on weekdays in the general population [18,21]. While weekend admissions are typically lower in absolute terms, our data did not reveal a significant difference in the volume of COVID-19 presentations between weekdays and weekends. A potential explanation for the higher admission rate on weekends may lie in the heightened clinical caution by ED clinicians during the early surge of the pandemic. At that time, limited understanding of the disease, absence of standardized treatment protocols, and uncertainty regarding the safety and reliability of outpatient follow up may have contributed to a lower threshold for hospitalization, particularly during weekends when access to ambulatory care resources was more constrained.

The existing literature on in-hospital mortality associated with weekend admissions yields mixed findings [22]. While several studies support a significant ‘weekend effect’, citing higher mortality rates for weekend admissions [14,23,24,25,26,27], others have reported no association [28,29] and some have even identified elevated mortality during mid-week admissions [30]. Similarly, in the context of COVID-19, available data are inconsistent, one study reported increased weekend mortality [31], whereas other showed a trend toward reduced mortality [32]. Our findings align with studies refuting a weekend mortality effect. However, these results must be interpreted with caution, as they are likely influenced by diagnosis-specific factors, institutional practice, and unique circumstance of the early pandemic period during which clinical uncertainty was high and standardized treatment protocols were evolving. Additional limitations include the exclusion of out-of-hospital deaths, potential misclassification of COVID-19 related mortality, and our study analysis of a single healthcare system. Furthermore, the heterogeneity in case-mix adjustment methodologies across prior studies presents challenges to direct comparison and meta-analytic comparisons [22]. Although weekend staffing shortages and limited weekend resources have also been hypothesized as contributing factors, though robust evidence remains limited [15].

In this study, three independent predictors of mortality were identified among patients presenting to ED with COVID-19 infection: absence of a primary care provider (PCP), hypoxia at presentation, and hyperglycemia on admission. During the early phase of pandemic, widespread outpatient clinics closure significantly limited access to primary care, potentially contributing to delayed treatment and likely increased disease severity at the time of ED presentation [18,21,33,34]. Additionally, the lack of available targeted antiviral therapies during this period further constrained outpatient management options. Hypoxia on admission, defined as peripheral oxygen saturation (SpO_2_) <95%, has consistently been associated with worse outcomes in COVID-19 and forms the basis of several predictive models [35,36,37,38,39]. Our findings reinforce this well-established association. Similarly, admission hyperglycemia has been shown to correlate with increased mortality, prolonged hospitalization, and progression to ARDS [40,41]. Glycemic variability and stress hyperglycemia are also known mortality predictors in the general population [42,43,44], and hyperglycemia has been repeatedly linked to adverse outcomes in COVID-19 [45,46,47]. Our study contributes further to this body of evidence. Interestingly, we also observed that the presence of crackles on lung auscultation was associated with lower mortality. While this finding is notable, it should be interpreted with caution given the subjective nature of physical examination and potential variability in auscultatory assessments across clinicians.

Several limitations of this study merit consideration. First, as a retrospective analysis, the findings are inherently dependent on the accuracy and completeness of data documented in the electronic medical record, which may be subject to misclassification or omission bias. Second, the study was conducted within a single healthcare system encompassing five hospitals located in the same geographic region, potentially limiting the generalizability. Third, although multivariable models adjusted for a range of clinical and sociodemographic factors, residual confounding by unmeasured or unrecorded factors cannot be excluded. Fourth, the data reflect a unique period, the first three months of the COVID-19 pandemic, when no effective treatments or vaccines were available. As such, the findings may not apply to later phases of the pandemic or to infections with subsequent SARS-CoV-2 variants. Finally, our study does not include deaths occurring outside the hospital, potentially underestimating true mortality rates.

## 5. Conclusions

In summary, this study found that patients with COVID-19 infection presenting to the ED on weekends were more likely to be admitted, however, no significant difference in mortality was observed based on the day of presentation. Notably, the absence of a primary care physician, hypoxia at presentation, and admission hyperglycemia emerged as independent predictors of mortality. These findings underscore the influence of clinical uncertainty during the early phase of the pandemic on admission decisions and highlight the prognostic significance of readily identifiable clinical variables. Future studies should evaluate whether these trends persist across subsequent phases of pandemic and within diverse healthcare systems.

## Figures and Tables

**Table 1:** Characteristics of study population.

Characteristics ^◊^	Weekend (*n* = 685)	Weekday (*n* = 2082)	*p*-Value *
Age in years, mean (SD)	55.7 (19)	54.8 (19)	0.29
BMI kg/m^2^ ≥ 30, *n* (%)	222 (39)	671 (39)	0.96
Female, *n* (%)	306 (45)	957 (46)	0.57
Race			0.64
White, *n* (%)	185 (28)	534 (26)	
African American, *n* (%)	217 (32)	698 (34)	
Others (Asian, American Indian, American native Hawaiian or Pacific Island), *n* (%)	267 (40)	807 (40)	
Ethnicity			
Hispanic or Latino, *n* (%)	225 (33)	682 (33)	0.96
Married or living with a partner, *n* (%)	211 (41)	704 (45)	0.17
Employment status, *n* (%)			0.71
Employed	256 (40)	765 (40)	
Unemployed, with disability or unable to work	341 (53)	1,032 (54)	
Retired, student, homemaker	42 (7)	109 (6)	
Uninsured, *n* (%)	109 (17)	359 (18)	0.41
No primary care physician, *n* (%)	311 (45)	876 (42)	0.13
Median ED length of stay in minutes, (IQR)	276 (162–413)	261 (146–395)	0.07 **
Mean hospital length of stay in days, (SD)	6.9 (10.6)	6.6 (10.2)	0.59
Cigarette use (current and ex-smoker vs. never), *n* (%)	142 (21)	433 (21)	>0.99
Alcohol use (current and past vs. never), *n* (%)	212 (31)	659 (32)	0.74
Reported exposure to COVID-19, *n* (%)	245 (36)	755 (36)	0.86
Reported recent travel, *n* (%)	18 (3)	93 (4)	**0.03**
Presented to ED from nursing home, inpatient rehab, group home, adult daycare, homeless, *n* (%)	109 (16)	307 (15)	0.46
COVID-19 tested more than once, *n* (%)	420 (61)	1249 (60)	0.56
Age adjusted Charlson comorbidity index (CCI) ^¥^ > 3, *n* (%)	287 (42)	839 (40)	0.47
Mean duration of symptoms at presentation in days, (SD)	5 (6)	5.6 (6.1)	**0.04**
Admitted to the hospitalized from ED, *n* (%)	440 (64)	1238 (59)	**0.03**
Mortality, *n* (%)	190 (28)	567 (27)	0.81

***^◊^*** For some patients the variables had missing value; * Chi-Square, Fisher’s exact statistic (where at least 20% of frequencies were <5) and unpaired *t*-test statistic (where presenting value was mean); ** Mann–Whitney U test; **^¥^** Charlson comorbidity index (CCI)—scores of 0, 1, 2, and 3 predicting 10-year survival rates of 93%, 73%, 52%, and 45%, respectively. The boldface type indicates statistical significance. BMI, body mass index; SD, standard deviation; IQR, interquartile range.

**Table 2:** Clinical symptoms, signs, and vitals statistics of study population.

Variables ^◊^	Weekend (*n* = 685)	Weekday (*n* = 2082)	*p*-Value *
Symptoms at presentation			
Fever, *n* (%)	414 (60)	1323 (64)	0.15
Chills, *n* (%)	161 (24)	559 (27)	0.09
Dry cough, *n* (%)	351 (51)	1099 (53)	0.51
Productive cough, *n* (%)	57 (8)	169 (8)	0.87
Nasal congestion, n (%)	48 (7)	153 (7)	0.80
Rhinorrhea, *n* (%)	23 (3)	62 (3)	0.61
Conjunctival irritation, *n* (%)	3 (0.4)	4 (0.2)	0.38
Sore throat, *n* (%)	93 (14)	330 (16)	0.16
Dysphagia, *n* (%)	5 (0.7)	5 (0.2)	0.08
Shortness of breath, *n* (%)	330 (48)	947 (46)	0.23
Hemoptysis, *n* (%)	7 (1)	15 (0.7)	0.46
Myalgia, *n* (%)	217 (32)	731 (35)	0.10
Fatigue, *n* (%)	175 (26)	491 (24)	0.30
Headache, *n* (%)	97 (14)	352 (17)	0.10
Anorexia, *n* (%)	14 (2)	39 (2)	0.75
Loss of smell, *n* (%)	35 (5)	119 (6)	0.63
Alteration in taste sensation, *n* (%)	42 (6)	168 (8)	0.11
Diarrhea, *n* (%)	89 (13)	288 (14)	0.61
Nausea, *n* (%)	48 (7)	133 (6)	0.59
Vomiting, *n* (%)	72 (11)	190 (9)	0.29
Appetite loss, *n* (%)	47 (7)	152 (7)	0.73
Chest pain, *n* (%)	96 (14)	302 (15)	0.80
Abdominal pain, *n* (%)	46 (7)	160 (8)	0.45
Generalized weakness, *n* (%)	81 (12)	233 (11)	0.68
Dizziness, *n* (%)	34 (5)	102 (5)	0.92
Seizure, *n* (%)	2 (0.3)	13 (0.6)	0.38
Syncope, *n* (%)	56 (8)	154 (7)	0.51
Clinical signs at presentation to ED			
Frail, *n* (%)	106 (15)	257 (12)	**0.04**
Dry oral mucosa, *n* (%)	64 (9)	139 (7)	**0.02**
Poor orodental hygiene, *n* (%)	3 (0.4)	7 (0.3)	0.72
Conjunctival injection, *n* (%)	2 (0.3)	8 (0.4)	>0.99
Throat congestion, *n* (%)	2 (0.3)	10 (0.5)	0.74
Tonsillar swelling, *n* (%)	0 (0)	2 (0.1)	1.00
Lymphadenopathy, *n* (%)	1 (0.1)	1 (0.1)	0.43
Respiratory distress, *n* (%)	135 (20)	335 (16)	**0.03**
Hypoxia, *n* (%)	123 (18)	308 (15)	**0.05**
Wheezing, rhonchi or coarse breath sounds, *n* (%)	85 (12)	219 (11)	0.81
Crackles, *n* (%)	52 (8)	114 (5)	**0.05**
Elevated jugular venous pressure (JVP), *n* (%)	8 (1)	22 (1)	0.83
Tenderness on examination, *n* (%)	36 (5)	98 (5)	0.54
Facial drool, *n* (%)	1 (0.2)	5 (0.2)	>0.99
Angle of mouth deviation, *n* (%)	0 (0)	1 (0.1)	>0.99
Motor weakness, *n* (%)	15 (2)	23 (1)	0.06
Sensory loss, *n* (%)	3 (0.4)	7 (0.3)	0.72
Tremors, *n* (%)	5 (0.7)	14 (0.7)	0.80
Edema, *n* (%)	26 (4)	67 (3)	0.47
Unresponsive, *n* (%)	17 (3)	47 (2)	0.77
Vitals at initial ED encounter			
Systolic blood pressure, mean (SD)	131 (22)	132 (22)	0.17
Diastolic blood pressure, mean (SD)	77 (15)	78 (14)	0.61
Heart rate, mean (SD)	95 (18)	95 (19)	0.76
Mean arterial pressure, mean (SD)	95 (16)	96 (15)	0.22
Temperature, mean (SD)	37.4 (0.87)	37.4 (0.86)	0.36
Respiratory rate per minute, mean (SD)	21 (6)	20 (6)	**0.02**
Peripheral oxygen saturation SpO_2_, mean (SD)	95 (4.5)	96 (4.9)	0.50
Use of oxygen at home prior to admission, *n* (%)	7 (1)	17 (0.8)	0.64
FiO_2_%, mean (SD)	25 (14.3)	25 (13.4)	0.48
Mortality, *n* (%)	190 (28)	567 (27)	0.81

***^◊^*** For some patients, the variables had missing value; * Chi-Square, Fisher’s exact statistic (where at least 20% of frequencies were <5) and unpaired *t*-test statistic (where presenting value was mean); The boldface type indicates statistical significance.

**Table 3:** Common admission diagnoses, and laboratory and imaging workup.

Variables ^◊^	Weekend (*n* = 685)	Weekday (*n* = 2082)	*p*-Value *
Common primary diagnosis at presentation			
Acute respiratory failure, *n* (%)	182 (27)	489 (23)	0.11
Pneumonia, *n* (%)	287 (42)	786 (38)	0.06
Pulmonary embolism, *n* (%)	5 (0.7)	16 (0.8)	>0.99
Congestive heart failure, *n* (%)	14 (2)	27 (1)	0.20
Sepsis, *n* (%)	44 (6)	106 (5)	0.21
COPD exacerbation, *n* (%)	9 (1)	25 (1)	0.84
Deep venous thrombosis, *n* (%)	2 (0.3)	7 (0.3)	>0.99
Cerebrovascular event, *n* (%)	3 (0.4)	3 (0.1)	0.17
Acute renal failure, *n* (%)	29 (4)	113 (5)	0.23
Acute bronchitis, *n* (%)	30 (4)	78 (4)	0.50
Unresponsive, *n* (%)	17 (3)	47 (2)	0.77
Laboratory work up at admission			
Hemoglobin, mean (SD)	13 (2.4)	13 (2.2)	0.46
White blood cell count, mean (SD)	8 (6.7)	7.7 (5.9)	0.34
Absolute neutrophil count, mean (SD)	5.7 (3.7)	5.8 (4.3)	0.59
Absolute lymphocyte count, mean (SD)	1.5 (3.9)	1.3 (3.9)	0.35
Neutrophil to lymphocyte count ratio ≥ 3, *n* (%)	371 (70)	1,058 (71)	0.50
Platelet count, mean (SD)	214 (83)	220 (91)	0.20
Erythrocyte sedimentation rate, mean (SD)	57.3 (34)	61.3 (35)	0.28
Serum ferritin, median (IQR)	657 (300–1215)	637 (313–1151)	0.82
Sodium, mean (SD)	137 (5.8)	137 (5.9)	0.94
Creatinine, mean (SD)	1.44 (2)	1.48 (2)	0.73
Glomerular fraction rate, mean (SD)	77 (34)	76 (34)	0.38
Glucose, mean (SD)	141 (71)	145 (76)	0.29
Chloride, mean (SD)	100 (6)	100 (6.5)	0.77
Potassium, mean (SD)	4.1 (0.6)	4.1 (0.6)	0.55
Hemoglobin A1c, mean (SD)	7.3 (1.9)	7.4 (2.2)	0.42
Aspartate aminotransferase (AST), mean (SD)	59 (133)	54 (113)	0.45
Alanine aminotransferase (ALT), mean (SD)	44 (58)	44 (160)	0.99
Alkaline phosphatase, mean (SD)	93 (72)	91 (54)	0.58
Albumin, mean (SD)	3.7 (0.6)	3.8 (0.7)	**0.02**
Calcium, mean (SD)	8.9 (0.7)	8.9 (0.7)	0.58
Total bilirubin, mean (SD)	0.59 (0.56)	0.55 (0.51)	0.20
C-reactive protein (CRP), median (IQR)	11.8 (3.9–36.8)	11.1 (4.5–34.2)	0.90 **
Lactate dehydrogenase (LDH), median (IQR)	343 (255–477)	354 (257–509)	0.55 **
Creatinine Phosphokinase (CPK), median (IQR)	127 (74–284)	129.5 (62–281)	0.56 **
Positive troponin, *n* (%)	81 (20)	227 (20)	>0.99
Pro-B-type natriuretic peptide (Pro-BNP), median (IQR)	205 (53–1181)	190 (44–947)	0.27 **
INR, mean (SD)	1.16 (0.64)	1.17 (0.65)	0.67
D-dimer, mean (SD)	1.92 (3.8)	2.5 (5.1)	0.06
Prothrombin time (PT), mean (SD)	11.6 (3.6)	12 (5.7)	0.29
Activated partial thromboplastin time (APTT), mean (SD)	33 (13.1)	32 (8.5)	0.18
Interleukin-6 (IL6), median (IQR)	61 (24.4–158.3)	59.6 (23.4–151)	0.97 **
Procalcitonin high-risk, *n* (%)	63 (27)	180 (28)	0.73
Type and Screen ordered, *n* (%)	203 (30)	567 (27)	0.24
Blood culture collected, *n* (%)	305 (45)	816 (39)	**0.02**
Positive Blood culture, *n* (%)	28 (9)	59 (7)	0.32
Urine culture collected, *n* (%)	117 (17)	318 (15)	0.28
Urine culture positive, *n* (%)	41 (71)	107 (70)	>0.99
Sputum culture collected, *n* (%)	19 (2.8)	43 (2)	0.30
Sputum culture positive, *n* (%)	9 (47)	23 (54)	0.78
Comprehensive viral panel, *n* (%)	25 (4)	89 (4)	0.51
Comprehensive viral panel positive, *n* (%)	1 (3.8)	1 (1)	0.40
QTc > 500 milliseconds, *n* (%)	41 (9)	99 (8)	0.49
30-day readmission to the same health care system, *n* (%)	69 (10)	187 (9)	0.40
Imaging work up at admission ***			
Duplex studies done at extremities to r/o DVT, *n* (%)	21 (3)	43 (2)	0.14
CT head and neck with abnormality, *n* (%)	9 (14)	39 (18)	0.57
CT Chest with abnormality, *n* (%)	78 (90)	323 (88)	0.85
CTA chest with abnormality, *n* (%)	6 (17)	24 (21)	0.81
CT abdomen pelvis with abnormality, *n* (%)	24 (48)	72 (43)	0.63
Chest x-ray (CXR) with abnormal read, *n* (%)	358 (68)	1035 (68)	0.87
Echocardiography with abnormal read, *n* (%)	11 (21)	41 (26)	0.47
Ultrasound abdomen with abnormal read, *n* (%)	6 (30)	11 (28)	>0.99
MRI brain with abnormal read, *n* (%)	3 (60)	8 (38)	0.62
MRI abdomen pelvis with abnormal read, *n* (%)	1 (100)	1 (33)	0.99

***^◊^*** For some patients, the variables had missing value; * Chi-Square, Fisher’s exact statistic (where at least 20% of frequencies were <5) and unpaired *t*-test statistic (where presenting value was mean); ** Mann–Whitney U test; *** Frequency for abnormal imaging studies calculated from the total number of imaging ordered (therefore denominator for each frequency and percentage is different). The boldface type indicates statistical significance. CT, computer tomography; CTA, computer tomography angiography; MRI, magnetic resonance imaging; SD, standard deviation; IQR, interquartile range.

**Table 4:** Predictors of mortality for patients presenting to ED with COVID-19 positive infection.

Risk Factors Analysis	Odds Ratio (95% CI)	
Socioeconomic predictors	Unadjusted Model ^1^*	Adjusted Model ^2^*
Age ≥ 55 years	**1.21 (1.02–1.43)**	**1.47 (1.01–2.15)**
Uninsured	1.20 (0.96–1.49)	**2.05 (1.25–3.37)**
No primary care provider	**1.65 (1.40–1.96)**	**1.73 (1.32–2.26)**
Reported exposure to COVID-19	**0.74 (0.62–0.88)**	**0.72 (0.53–0.96)**
Presentation symptoms predictors	Unadjusted Model *	Adjusted Model ^3^*
Chills	1.16 (0.96–1.40)	**1.40 (1.14–1.73)**
Dry cough	**0.73 (0.61–0.86)**	**0.71 (0.59–0.87)**
Productive cough	0.84 (0.61–1.16)	**0.70 (0.50–0.99)**
Sore throat	1.15 (0.92–1.45)	**1.32 (1.04–1.68)**
Myalgia	**0.78 (0.66–0.94)**	**0.81 (0.66–0.98)**
Presentation signs predictors	Unadjusted Model *	Adjusted Model ^4^*
Frail	1.25 (0.99–1.59)	**1.35 (1.03–1.76)**
Crackles	**0.52 (0.24–0.79)**	**0.52 (0.34–0.79)**
Presentation vital statistics predictors	Unadjusted Model *	Adjusted Model ^5^*
Respiratory rate; <20 per minute vs. ≥20 per minute	0.86 (0.72–1.02)	**0.72 (0.59–0.87)**
Pulse oxygen: SpO_2_ level < 95% vs. ≥ 95%	**1.39 (1.16–1.68)**	**1.53 (1.25–1.87)**
Presentation laboratory work up predictors	Unadjusted Model *	Adjusted Model ^6^*
Hematology		
Neutrophil to lymphocyte count ratio ≥ 3	1.19 (0.96–1.48)	**1.38 (0.50–2.71**)
Serum chemistry	Unadjusted Model *	Adjusted Model ^7^*
Hyperglycemia (Glucose > 100 mg/dL vs. 71–100 mg/dL)	**1.62 (1.26–2.08)**	**5.59 (1.18–26.4)**
Potassium millimoles per liter (mmol/L)	**1.22 (1.04–1.44)**	**2.64 (1.13–6.14)**
Creatinine Phosphokinase (CPK), units per liter < 165–232 vs. >165–232	1.34 (0.98–1.83)	**0.37 (0.15–0.91)**
C-reactive protein (CRP) mg/dL	1.00 (0.99–1.00)	**0.98 (0.97–0.99)**
Final adjusted model		Adjusted Model ^8^*
No primary care provider		**3.47 (2.37–5.07)**
Crackles		**0.47 (0.24–0.92)**
Pulse oxygen: SpO_2_ level < 95% vs. ≥5%		**1.46 (1.001–2.12)**
Hyperglycemia (Glucose > 100 mg/dL vs. 71–100 mg/dL)		**2.13 (1.25–3.65)**

* Unadjusted model for individual variables. ^2^* Adjusted model for socioeconomic risk factors thought to influence mortality (age, gender, race, ethnicity, marital status, employment status, having insurance, having a primary care provider, smoking status, alcohol use, presenting from nursing home/inpatient rehab/group home/adult daycare or homeless, reported exposure to COVID-19, reported recent travel, obesity, weekend admission, and Charlson comorbidity index). ^3^* Adjusted model for clinical symptoms including fever, chills, dry cough, productive cough, nasal congestion, rhinorrhea, sore throat, dysphagia, shortness of breath, hemoptysis, myalgia, fatigue, headache, anorexia, loss of smell, alternation in taste sensation, diarrhea, nausea, vomiting, loss of appetite, chest pain, abdominal pain, generalized weakness, dizziness and seizure. ^4^* Adjusted model for signs at presentation including, frail, dry oral mucosa, poor orodental hygiene, conjunctival injection, throat congestion, lymphadenopathy, respiratory distress, hypoxia, wheezing, rhonchi or coarse breath sounds, crackles, elevated jugular venous pressure, tenderness on examination, facial drool, motor weakness, sensory loss, tremors, edema and unresponsiveness. ^5^* Adjusted model for vital signs, including systolic and diastolic blood pressure, heart rate, temperature, respiratory rate, and pulse oxygen level. ^6^* Adjusted model for initial hematology laboratory work up including hemoglobin, neutrophil to lymphocyte count ratio, platelet count, erythrocyte sedimentation rate, ferritin, d-dimer and hemoglobin A1c levels. ^7^* Adjusted model for initial chemistry laboratory work up including, sodium, GFR, glucose, chloride, potassium, AST, ALT, Alk phos, albumin, calcium, troponin levels, creatinine phosphokinase, lactate dehydrogenase, C-reactive protein, pro-B-type natriuretic peptide, interleukin-6 and procalcitonin. ^8^* Final adjusted model for all statistically significant variables in above categories including, age, having insurance, having a primary care provider, reported exposure to COVID-19, chills, dry cough, productive cough, sore throat, frail, myalgia, crackles, respiratory rate, pulse oxygen level, neutrophils to lymphocyte count ratio, hyperglycemia, potassium levels, creatinine phosphokinase, and pro-B-type natriuretic peptide Boldface type indicates statistical significance. CI, confidence interval.

## Data Availability

The original contributions presented in this study are included in the article. Further inquiries can be directed to the corresponding author.
